# Teleoncology: Novel Approaches for Improving Cancer Care in North America

**DOI:** 10.7759/cureus.43562

**Published:** 2023-08-16

**Authors:** Adam J Elder, Hussein Alazawi, Fareshta Shafaq, Adam Ayyad, Ribhi Hazin

**Affiliations:** 1 Department of Medical Education, Wayne State University School of Medicine, Detroit, USA; 2 Department of Medical Education, Michigan State University College of Osteopathic Medicine, East Lansing, USA; 3 Department of Medical Education, American University of the Caribbean, Cupecoy, SXM; 4 Department of Medical Education, Ross University School of Medicine, Bridgetown, BRB; 5 Department of Internal Medicine, Wayne State University School of Medicine, Detroit, USA

**Keywords:** neuro-oncology, improving health outcomes, underserved patients, telehealth, telesurgery, pediatric oncology, telemedicine, teleoncology

## Abstract

Due to widespread healthcare workforce shortages, many patients living in remote and rural North America currently have reduced access to various medical specialists. These shortages, coupled with the aging North American population, highlight the need to transform contemporary healthcare delivery systems. The exchange of medical information via telecommunication technology, known as telemedicine, offers promising solutions to address the medical needs of an aging population and the increased demand for specialty medical services. This progressive movement has also improved access to quality health care by mitigating the current shortage of trained subspecialists. Minimizing the effects of these shortages is particularly urgent in the care of cancer patients, many of whom require regular follow-up and close monitoring. Cancer patients living in remote areas of North America have reduced access to specialized care and, thus, have unacceptably high mortality and morbidity rates. Teleoncology, or the use of telemedicine to provide oncology services remotely, has the ability to improve access to high-quality care and assist in alleviating the burden of some of the severe adverse events associated with cancer. In this review, the authors describe how recent advances in teleoncology can reduce healthcare disparities and improve future cancer care in North America.

## Introduction and background

Introduction: disparities in health care

Despite recent medical advances, shortages of trained specialists exist throughout North America, where as many as one in seven citizens remains "medically underserved" [1]. Most medical subspecialists remain clustered in urban centers serving as a referral center for a large geographic area and population. Individuals in rural or remote North America lack easy access to these referral centers and must travel long distances to receive subspecialty care. For individuals suffering from chronic diseases, such as cancer, the prospects are particularly bleak when one considers the fact that the most recent American Society of Clinical Oncology (ASCO) data anticipate a total shortage of 2,393 oncologists and radiation oncologists by 2025 as the increase in demand outgrows the rise in supply [2]. Although attempts to eliminate healthcare disparities are complex and often require governmental involvement, telemedicine may help avert the projected crisis by improving access to quality health care.

In the United States and other countries where most subspecialists are clustered in urban settings [3], individuals living in remote areas disproportionately suffer the burden of chronic diseases like cancer [4] and have unacceptably high mortality and morbidity rates associated with these diseases [5,6]. This is mainly due to the disproportionate quality of care provided to these patients. The current literature is replete with instances of international telemedicine-based twinning programs that have positively impacted survival and outcomes in low-income nations [7-11]. However, little has been written about technology's potential impact in developed countries like the United States and Canada, where individuals living in remote locations are often forced to accept suboptimal medical treatment due to reduced access to regional urban healthcare facilities. The following overview will focus on how advances in technology and recent experiences in international teleoncology can help address the maldistribution and shortage of specialists in remote areas of North America and, as a result, enhance the provision of cancer care on the continent.

Disparities in cancer care in North America

In the United States, where approximately 25% of individuals live in rural areas [12,13], and in Canada, where 21.5% of citizens live in remote locations [14], cancer patients often face unique challenges in accessing adequate cancer care services. A recent report by the Canadian Institute for Health Information revealed that Canadians living in remote areas, many of whom require 120 miles of travel or more to reach the nearest specialist [14], were "less healthy" than their urban and suburban counterparts [14]. Managing cancer or other chronic diseases with a multi-specialty approach can represent a severe logistical challenge in a nation where many regions lack trained pathologists, oncologists, and other specialists. In addition to cancer care, patients living in remote areas throughout North America also have reduced access to many different types of specialized health care.

Similarly, a recent study in North Carolina found that Americans living in rural areas were among the "most unhealthy" in the United States [15,16] and had the worst death rates in the region from chronic diseases [16]. In addition to geographical separation, inaccessibility to specialized health care can also be influenced by economic considerations, reduced mobility, incarceration, and social marginalization [17-19]. Given North America's current shortage of nurses, this situation is expected to worsen for cancer patients who rely on nurse-intensive long-term treatment programs such as hospice care and home health care. According to a report from January 2023, long-term care facilities throughout the United States have lost 210,000 jobs over the course of the pandemic from February 2020 to December 2022 [20]. As the current nursing shortage in the US is not dire enough, experts estimate there will be a shortage of 260,000 registered nurses by 2025 [21].

## Review

Telemedicine

Telemedicine, or exchanging medical information via telecommunication technology, permits rapid consultation between healthcare professionals in remote locations. Such novel strategies offer promise in addressing the medical needs of an aging North American population, the increased demand for specialty medical services, and the current shortage of trained subspecialists.

Infrastructure

The infrastructure required for telemedicine involves a computer and a high-speed internet connection capable of transferring data and images rapidly. Depending on the application or needs of healthcare collaborators, specific digitized peripheral components such as a video camera or digital diagnostic equipment (i.e., stethoscope, otoscope, X-ray, and magnetic resonance imaging equipment) can be added to this essential infrastructure to facilitate real-time video teleconferencing, the transfer of pathological or radiological images, or any other medical data that participants deem necessary to their proposed consultation [6,18]. Such instrumentation is readily accessible in today's information-based society [18].

Telemedicine in Practice

By improving access to specialized medical care, telemedicine can enhance patient convenience and, thus, positively impact patient compliance and continuity of care [22].

Telemedicine has been used, in varying capacities, in nearly every field of medicine, including but not limited to radiology [23,24], psychiatry [25], dermatology [26], pathology [27], cardiology [22], endocrinology [28], surgery [29,30], gastroenterology [31], ophthalmology [32], oncology [7,13], and pain management [33]. By leveraging existing technology, telemedicine allows clinicians separated by geographic distance to collaborate on any combination of the following components of medical management, including telediagnosis, teleconsultation, telepathology, telesurgery, and tele-follow-up (Table 1).

**Table 1:** Various components of telemedicine Adapted from [12].

Telemedical terminology
Telediagnosis: The utilization of computers and sophisticated video and audio modalities to facilitate the examination of patients in remote localities.
Teleconsultation: The process of allowing a multidisciplinary team to meet via video teleconferencing to discuss cases and guide the management of patients in distant areas.
Telepathology: The practice of transferring pathological specimens or images to a distant pathologist for evaluation.
Telesurgery: The use of technology to consult distant surgical specialists on modes of surgical treatment for improving outcomes of local cases.
Teleradiotherapy: Describes the use of the internet and digital technology to enhance access to radiotherapy for patients living in remote regions.
Tele-follow-up: The practice of harnessing telemedicine technology to monitor disease progression and treatment results in patients completing therapy.

Although traditional diagnosis relies upon obtaining a detailed patient history and comprehensive physical examination in person, telemedicine allows local healthcare professionals to conduct physical inspections and transmit their findings to the consultant. These interactions often depend on computers to enhance images and audio-visual files from precision cameras, electronic stethoscopes, and other digitized diagnostic medical devices [34,35]. The findings of telediagnosis can be digitized, stored, and shared via an internet connection and ultimately used in teleconsultations. Teleconsultation involves multidisciplinary medical teams meeting via video teleconferencing to help facilitate the formalization of management strategies.

Teleoncology

Teleoncology in Bridging International Healthcare Disparities

One aspect of telemedicine that has proven effective is teleoncology, or the delivery of oncology services from a distance to facilitate consultation by cancer specialists to geographically remote primary care providers [36,37]. Since accurate pathological evaluation remains a cornerstone of cancer treatment in remote locations where experienced pathologists are often difficult to access, telepathology can be vital for diagnostic accuracy [27,35]. Although the physical transfer of intraoperative or biopsy specimens to a distant site is often necessary for telepathology, much of the interaction between pathologists and local care teams can be undertaken via teleconsultation. The impact of such telemedicine collaborations can be maximized by input from medical oncologists, radiation oncologists¸, surgeons, social workers, and others involved in the care of individual patients [36]. This can be especially useful for including primary care physicians in patient treatment planning and ultimately providing the highest quality care for a shared patient. Using telemedicine to improve local delivery capacity in remote healthcare facilities and increase online physician consultations with cancer patients can improve cancer care and patient satisfaction [38,39].

Teleoncology has proven effective in improving cancer care globally by allowing low-income nations with limited resources and few trained medical experts to tap into the vast reservoir of resources available in developed countries. In low-income nations where adequate medical infrastructure is typically lacking [3,17] and where the number of trained specialists is "woefully inadequate" [3], teleoncology provides a reliable method of linking healthcare specialists regardless of geographic separation [36,40]. This is especially true in oncology subspecialties such as neuro-oncology, as the mortality in these subgroups can be as high as 90% in economically disadvantaged countries compared to approximately 4% mortality in well-developed nations [25].

In oncology, where multidisciplinary management has been recognized as a critical aspect of improving cancer outcomes [36,40], teleoncology offers low-income nations and individuals living in remote areas a flexible means of adopting a multidisciplinary treatment approach to managing and treating cancer [40]. A recent collaboration between St. Jude Children's Research Hospital in Memphis, Tennessee, USA, and national healthcare institutions in Honduras and Guatemala used teleoncology to facilitate the development of early diagnosis programs for retinoblastoma and other central nervous system tumors [8]. Another series of similar mentorship programs between specialists at St. Jude Children's Research Hospital and physicians at the King Hussein Cancer Center in Amman, Jordan helped improve survival amongst patients with retinoblastoma [41] and medulloblastoma [7]. In these and other collaborative teleoncology initiatives [7-11], physicians and patients in low-income nations were given access to state-of-the-art technology, equipment, and expert consultation that they would not have otherwise accessed.

Teleoncology for Enhancing Cancer Screening and Early Detection

Given the sparse distribution of specialists in North America, establishing a multi-specialty management team represents one of many challenges facing cancer care providers in rural and remote locations. Screening and early detection have also been traditionally inadequate in underserved areas throughout North America [42-44]. The lack of early detection programs has been linked to cancer mortality rates as high as 30% in some remote areas of the United States [45]. Regular cancer screening can reduce cancer-related premature mortality and therapy-associated morbidity by detecting cancer at an earlier, more curable stage [44]. In the absence of teleoncology, such screening is arduous in remote locations. Most of the commonly used screening methods, such as mammography, colonoscopy, Papanicolaou (pap) smear, prostate-specific antigen (PSA), skin biopsy, and other pathology tests, can be accurately interpreted via teleoncology [42,44,46-48]. A recent initiative in Arizona successfully used teleoncology to screen immigrant, Hispanic women, and Native American women in remote locations for cervical and breast cancer [44,49]. After locally trained healthcare providers performed mammography, colonoscopy, and slide preparation, digital images of the slides and mammograms were instantaneously transmitted to a tertiary-care facility in Tucson, which generated an online report that included interpretation, eligibility for ongoing clinical trials, treatment options, online scheduling of real-time consultation, and a follow-up plan. Similar screening programs have since been implemented worldwide [42,47].

Because approximately 10-15% of all breast cancers are believed to be hereditary, and nearly 30% of these cases result from familial BRCA1 or BRCA2 mutations [46,47,50], cancer genetic counseling is widely recognized as a "standard of care" in at-risk women [24]. However, the cost and complexity of gene sequencing make screening widely unavailable [24,50], and genetic counselors, who can interpret and integrate the information, are rare in underserved areas [24]. For example, only two cancer genetic counselors serve rural parts of North Carolina, each practicing only a few days per month [24]. However, in a clinical trial currently underway at Duke University Medical Center (Durham, North Carolina), teleoncology is being utilized cost-effectively to provide quality genetic counseling in these areas by allowing patients to communicate with genetic specialists via videoconferencing [24]. Although teleoncology-based cancer screening remains unavailable mainly in developing countries, its growing application in the United States will likely serve as a paragon for other nations to emulate.

The COVID-19 Pandemic's Effect on Teleoncology

The COVID-19 pandemic has redefined healthcare delivery, with telemedicine surging to the front as a pivotal solution. As medical buildings such as hospitals and clinics became potential hotspots for virus transmission, we intensified the need to protect vulnerable populations such as cancer patients. Telemedicine ensured continuity of care and established a framework that the world could utilize in the face of future pandemics that limit face-to-face contact. Some of the most significant merits of teleoncology during the pandemic were its ability to offer remote consultations to safeguard immunocompromised cancer patients from potential exposures, as well as alleviating the logistical and financial burdens associated with frequent hospital visits, especially for those residing in remote areas [51]. The lessons learned from the pandemic era can inform best practices, ensuring that teleoncology remains a sustainable and effective care model, even post-pandemic.

One crucial factor to consider in teleoncology is the triage of cancer patients, establishing the need for immediate consultation or intervention based on the urgency of the patient's condition. Correct triage ensures that patients with aggressive or advanced-stage cancers and those exhibiting severe symptoms are prioritized. On the other hand, incorrect triage can lead to devastating consequences, including preventable disease progression and complications. Establishing transparent and evidence-based triage protocols is essential to maintain trust and ensure optimal patient outcomes.

Teleoncology for Continuing Medical Education Amongst Professionals and Improving Public Awareness

In supporting cancer services in remote areas, teleoncology can facilitate acquiring education, training, and continuing medical education (CME) credits for cancer specialists. Teleoncology also reduces unnecessary referrals, increases the efficiency of diagnosis, positively impacts patient care, and allows for closer scrutiny of overall medical services [12,34]. Moreover, teleoncology initiatives also aid in the identification of areas requiring improvement, allow for the attainment of outstanding professional expertise without the need for travel, and reinforce professional relationships by increasing the opportunities for collaborative publications amongst healthcare professionals working in different institutions [7,41,52,53]. This is critical because most clinicians who practice in remote areas are, like the patients they treat, typically isolated from essential educational opportunities and interactions with medical experts and subspecialists [53].

Ultimately, teleoncology represents a largely untapped resource that can upgrade existing health delivery services and improve the confidence level among remote primary care providers and expert oncologists [38,54]. This can be accomplished via digital video conferences and televised educational nursing courses created to hone the expertise of those involved in the care of local patients [55]. Patients and their advocates have also supported teleoncology programs since they prevent the need for disruptive and expensive trips to regional cancer centers. Teleoncology also allows cancer patients to receive valuable care in the presence of their family and other essential support networks that are often critical in terminally ill cancer patients [54,56]. Further, patients can derive invaluable supportive clinical care through assessments of pain and nutrition via teleoncology applications [55].

Teleoncology in Improving Radiotherapy

Radiotherapy remains a primary component of the integrated management of many cancers. However, establishing costly primary radiotherapy centers in remote areas with minimal budgetary allocations is often challenging [57]. In the few remote locations with adequate radiotherapy infrastructures and facilities, the lack of trained experts often represents a challenge in providing quality care to cancer patients [57]. By leveraging teleoncology resources, teleradiotherapy can help expand the availability of radiotherapy to patients in sparsely populated regions and reduce errors in the provision of treatment [57,58].

Teleradiotherapy can occur only after digital computed tomography (CT) and relevant magnetic resonance imaging (MRI) studies are digitized and sent to the main radiotherapy center. Once the main center receives these studies, appropriate radiotherapy strategies are formulated, adopted, and performed on the patient via a trained local operator [58]. Daily imaging and regular consultation between radiotherapy experts in urban centers and clinicians and technicians in remote locations can reduce positioning errors and increase beam utility in delivering radiation therapy to patients [59]. Notwithstanding, telemedicine has not sufficiently addressed certain limitations in providing access to radiotherapy. Despite recent advances, many highly effective, non-invasive radiotherapy modalities, such as intensity-modulated radiation therapy (IMRT), proton beam radiotherapy (PBRT), and Gamma Knife radiosurgery, remain mainly inaccessible to individuals living in remote or rural locations.

Teleoncology for Monitoring Chemotherapy

Advances in chemotherapy have enabled the delivery of chemotherapeutic treatments from a patient's home. Despite its convenience for patients, home-based chemotherapy can make it challenging to detect the adverse effects of therapy [60]. Cancer patients are now using teleoncology to keep regularly scheduled, web-based symptom diaries that are instantaneously sent to the pager or mobile phone of the clinician designated to monitor and archive these entries [60]. Leveraging telemedicine technology to enable this type of daily interaction between patient and healthcare coordinator (e.g., oncologist or nurse liaison) has proven effective in recognizing the adverse effects of chemotherapeutic agents in cancer patients [60]. Although telemedicine may not necessarily improve access to chemotherapeutic medications, recent pilot studies have demonstrated how pharmacists in large urban hospitals can use telemedicine to provide patients with chronic diseases in remote areas with round-the-clock pharmacist coverage that ensures safe and effective medication therapy [61]. After the chemotherapy is completed, teleoncology can be used for follow-up appointments.

Teleoncology for Promoting Clinical Trials

Future clinical trials and multi-center research would benefit immensely from teleoncology. Incarcerated, immobile, remote, or otherwise inaccessible patients could quickly be enrolled in clinical trials. Although real-time expert review of pathology and radiology images remains uncommon in hospitals, prospective use of such thoughts could improve patient outcomes by reducing diagnostic errors. Packer et al. performed a central radiology review at the end of a medulloblastoma study and found that patients who received inadequate radiological staging were less likely to survive [62]. If prospective teleradiology review had been integrated into the study, these patients' outcomes would presumably have been better. Another central pathology review at the end of a study found that 30% of patients diagnosed with high-grade glioma had low-grade glioma [63]. These patients underwent unnecessarily aggressive therapy, which the inclusion of a prospective telepathology review in the protocol could have prevented. Prompt feedback from experienced radiologists and pathologists via teleoncology could improve diagnostic accuracy and avoid suboptimal treatment of rare cancers. Teleoncology can also help to facilitate the inclusion of developing countries in clinical trials, as was recently demonstrated via a collaborative exchange between St. Jude Children's Research Hospital and oncologists in Chile [64]. Enhancing recruitment for clinical trials amongst traditionally underserved patients remains critical to decreasing cancer mortality rates in these populations [5].

Cost reduction and tele-follow-up

Telemedicine can significantly reduce the cost of healthcare delivery by mitigating provider shortages and preventing the need to travel long distances. Given the possibility of disease recurrence or remission in cancer patients, the need for tele-follow-up is critical for improving long-term outcomes. From a patient's perspective, the main barriers to follow-up are logistics and travel expenses [55,65].

In South Dakota, where many Native Americans live an average of 140 miles from the nearest cancer center, a recent study found that 47% of patients identified lack of transportation as a significant barrier to healthcare access [5]. A recent province-wide survey in Quebec, Canada examined the efficacy of telemedicine triage and revealed that 80% of individuals enrolled in the study saved an average of five hours and three hours of childcare costs, 60% avoided significant travel costs, and approximately 25% avoided lost time at their place of employment [66]. Further, previous studies have demonstrated how much time and money telemedicine programs can save physicians through their ability to reduce the number of unnecessary visits to emergency departments [67]. Teleoncology helps minimize the maldistribution of resources, the relevance of fuel costs, travel expenses, and other factors in the treatment of cancer patients and can encourage patients to seek regular follow-ups with the core care team in a manner that reduces the above inconveniences [65]. Maldistribution and the shortage of specialists in remote areas of North America have been summarized in Table 2.

**Table 2:** Examples of maldistribution of medical resources in North America

Medical shortage	Reference
One family physician per 3000 residents in some parts of Canada	[33]
Only one dermatologist in Nova Scotia (estimated population: 970,000)	[33]
Only two genetic cancer counselors serving all rural areas of North Carolina	[34]
Even though over 21% of children in the USA live in rural areas, only 3% of pediatric intensive care physicians practice in rural areas	[35]
Individuals in Hawaii cannot access specialty care without being flown into Honolulu	[36]
There are over 19,400 nursing vacancies (8.1% national vacancy rate) in long-term care facilities throughout the United States	[41]
American Indians in need of cancer treatment in South Dakota often travel an average of 140 miles to reach the nearest regional cancer facility	[66]

Potential drawbacks

The failure to aid physicians and ancillary staff in developing new skills can potentially hamper such initiatives and undermine quality assurance [23,56]. Training intended practitioners remains crucial in ensuring any telemedicine program's longevity. In addition to optimizing user performance and safety, well-trained technical staff can positively impact such initiatives and contribute positively to patient satisfaction with telemedicine applications [23,68,69]. Practitioners must express empathy and other emotions during telemedicine exchanges to promote patient satisfaction and strengthen doctor-patient relationships [68,69]. It is also crucial to recognize potential patient-related barriers to telemedicine. Technology access is a primary concern typically arising due to a lack of the required devices or stable internet access. Another challenge is being unfamiliar and lacking the knowledge to use the technology, which could be too demanding for older people. This digital divide accentuates the health disparities, where the most benefitting from telemedicine might be the least equipped to access it [70]. Another concern is privacy, as digital platforms carry an inherent risk of data breaches, making patients hesitant about sharing sensitive medical information online. We must also address the fear of compromised quality of care; virtual consultations lack the physical aspect of health care, which some conditions necessitate [70]. Finally, there is ambiguity regarding insurance coverage for telemedicine services, leading to potential out-of-pocket expenses. Without clear information on reimbursement, many might hesitate to choose virtual care.

Palliative care and end-of-life options remain inadequate in remote communities. Future research may be warranted to examine how telemedicine can improve the quality of and access to existing palliative care models in remote communities.

## Conclusions

By improving patient convenience and lowering costs, teleoncology can represent a novel approach for ensuring patient compliance, long-term follow-up, and improved access to quality cancer care in remote regions of North America. The success of such initiatives in the future hinges on the appropriate training of local practitioners and tailoring individual applications to the specific needs of patients and healthcare providers in each location. As we continue to confirm the success and significance of telemedicine in first-world countries such as the United States, we will be able to create the blueprint necessary to develop the same systems in other countries worldwide. The future of telemedicine is poised for unprecedented growth, as it emerges as a vital facet of health care. This provides a scalable solution as the world grapples with challenges like an aging population and limited access to health care in remote regions. However, its potential can only be fully realized with continuous research. It is imperative to address concerns related to data security, ensure equal access for all demographics, and validate new technologies for clinical efficacy. Continued research will solidify telemedicine's role, ensuring it remains patient-centric, efficient, and universally accessible. As telemedicine continues to evolve and advance, it can revolutionize healthcare delivery, reaching underserved areas and populations, thus bridging the gap in healthcare disparities and contributing to better health outcomes for all.

The future of telemedicine is poised for unprecedented growth, emerging as a vital facet of modern health care. With the integration of advanced technologies like artificial intelligence and machine learning, telemedicine will offer more accurate diagnostics, personalized treatments, and enhanced patient monitoring. Telemedicine provides a scalable solution as the world grapples with challenges like an aging population and limited access to health care in remote regions. However, its potential can only be fully realized with continuous research. Continued research will solidify telemedicine's role, ensuring it remains patient-centric, efficient, and universally accessible.
